# Training Data Selection and Optimal Sensor Placement for Deep-Learning-Based Sparse Inertial Sensor Human Posture Reconstruction

**DOI:** 10.3390/e23050588

**Published:** 2021-05-10

**Authors:** Zhaolong Zheng, Hao Ma, Weichao Yan, Haoyang Liu, Zaiyue Yang

**Affiliations:** 1 Shenzhen Key Laboratory of Biomimetic Robotics and Intelligent Systems, Department of Mechanical and Energy Engineering, Southern University of Science and Technology, Shenzhen 518055, China; 11930587@mail.sustech.edu.cn (Z.Z.); mah@sustech.edu.cn (H.M.); 12031235@mail.sustech.edu.cn (W.Y.); yangzy3@sustech.edu.cn (Z.Y.); 2 Guangdong Provincial Key Laboratory of Human-Augmentation and Rehabilitation Robotics in Universities, Southern University of Science and Technology, Shenzhen 518055, China; 3 School of Sports Engineering, Beijing Sport University, Beijing 100084, China

**Keywords:** Bi-RNN, Max-Relevance and Min-Redundancy, training data selection, pose estimation, optimal sensor placement

## Abstract

Although commercial motion-capture systems have been widely used in various applications, the complex setup limits their application scenarios for ordinary consumers. To overcome the drawbacks of wearability, human posture reconstruction based on a few wearable sensors have been actively studied in recent years. In this paper, we propose a deep-learning-based sparse inertial sensor human posture reconstruction method. This method uses bidirectional recurrent neural network (Bi-RNN) to build an a priori model from a large motion dataset to build human motion, thereby the low-dimensional motion measurements are mapped to whole-body posture. To improve the motion reconstruction performance for specific application scenarios, two fundamental problems in the model construction are investigated: training data selection and sparse sensor placement. The problem of deep-learning training data selection is to select independent and identically distributed (IID) data for a certain scenario from the accumulated imbalanced motion dataset with sufficient information. We formulate the data selection into an optimization problem to obtain continuous and IID data segments, which comply with a small reference dataset collected from the target scenario. A two-step heuristic algorithm is proposed to solve the data selection problem. On the other hand, the optimal sensor placement problem is studied to exploit most information from partial observation of human movement. A method for evaluating the motion information amount of any group of wearable inertial sensors based on mutual information is proposed, and a greedy searching method is adopted to obtain the approximate optimal sensor placement of a given sensor number, so that the maximum motion information and minimum redundancy is achieved. Finally, the human posture reconstruction performance is evaluated with different training data and sensor placement selection methods, and experimental results show that the proposed method takes advantages in both posture reconstruction accuracy and model training time. In the 6 sensors configuration, the posture reconstruction errors of our model for walking, running, and playing basketball are 7.25°, 8.84°, and 14.13°, respectively.

## 1. Introduction

Motion capture has a wide range of applications in medical rehabilitation, virtual reality, sports training and other fields [1,2,3]. However, the current inertial motion-capture devices require subjects to wear 17 or more inertial sensors [4]. It can be intrusive, time-consuming, and prone to sensor misplacement during mounting. Tracking with a minimal, lightweight configuration of sensors is therefore desirable. Many studies have shown that human motion includes a lot of redundant information and can be described by dimensions lower than the degree of freedom of human motion [5,6,7]. This opens the door to the study of using sparse inertial sensors human motion capture. To improve the ease of use of inertial motion-capture technology, in recent years researchers have begun to pay attention to motion-capture technology based on fewer wearable inertial sensors [8,9,10]. Existing research mainly focuses on motion reconstruction methods based on motion prior data and models. For example, a priori model of human motion including local posture linear regression models based on multilayer neural networks and deep-learning recurrent neural networks has been constructed, using 5–6 inertial sensor measurements as input to the model to predict the whole-body posture. Based on the attitude estimation of the prior model, von Marcard et al. merged the prior model with inertial measurements and proposed a motion reconstruction method with offline optimization.

At present, the research of motion-capture technology based on sparse inertial measurement is in the preliminary stage. Several motion prior models have been proposed for general motion capture, but the posture reconstruction accuracy is still limited. In addition, the existing research has not studied the influence of the position of the sparse inertial sensor on the reconstruction accuracy. The motivation of the work is to improve the accuracy of the sparse inertial sensor pose reconstruction, especially for certain target application scenarios. We believe that selecting appropriate training data and the optimal sensor placement on the human body are promising means to improve the accuracy of pose reconstruction under specific application scenarios.

Current methods mainly work in general purpose motion capture, where a large human motion dataset with various movements is used for model training. The basic assumption of training data for machine learning models is that the collected data should be independent and identically distributed (IID) in the target application scenario [11]. The model trained for general purpose may not be optimal for a specific application. Because the dataset is usually accumulated through many tests, it maybe imbalanced in different types of movements. The posture reconstruction accuracy has potential to be improved for certain applications where the movements are more regular and predictable. However, the practical challenge is that the existing motion-capture dataset may not fulfill the IID condition of a specific application scenario, and collecting large amount of IID data from each target application scenario can be time-consuming. To fully use existing dataset, we propose a new data selection method prior to training machine learning models according to the target application scenario. The general idea is that first we collect a small amount of IID reference data from the target scenario. Then, continuous data with small redundancy are selected from the existing dataset, so that they are complied with the a priori distribution of the reference data.

In addition to selecting appropriate training data, the placement of the sparse inertial sensors also plays an important role in the posture reconstruction. It is also worth studied for the optimal performance in different applications. For example, in normal gait, the thigh and shank sensor measurements have strong relevance, and it is possible to infer thigh posture according to the shank posture. In this case, these two sensors have large information redundancy. However, when playing football, the leg movement is highly random in a long period of time, where the independence between the thigh and shank measurements is larger. Generally, the relevance and independence among groups of sensors on the human body vary under different scenarios. The choice of sparse sensor placement will have an obvious impact on the body posture reconstruction accuracy. It is necessary to establish a model of the information volume related to the sensor configuration. With this model, we can determine the optimum number and placement positions of the sensors, and in this way, we can improve the accuracy of motion capture while ensuring the ease of use of the system.

In this paper, we first introduce a method to choose appropriate training data from the accumulated dataset, so that the selected data have similar distribution to data collected from the specific application scenario and has small redundancy. Then, the optimum number and placement position of the sparse inertial sensors are determined through information volume evaluation. Finally, the selected training data are used to train a deep-learning neural network, where the inputs are the motion measurements of the selected sensors and the output is the whole-body posture. The body posture reconstruction performance under different training data and sensor placements are evaluated through experiments. In summary, the novelty and contributions of this paper include:

- A novel optimization-based efficient data selection scheme is proposed to select continuous and IID data with small redundancy from a large dataset for a specific application scenario.
- The inertial sensor placement problem in sparse inertial sensor posture reconstruction is first formulated into an optimization problem, and a heuristic optimization method is proposed based on a metrics of the amount of information and the redundancy of a group of sensors.

The rest of this paper is organized as follows. Section 2 introduces related work. Section 3 provides overview of the proposed methods. Section 5 introduces the theory and algorithm of training data selection. Section 6 describes the correlation and the redundancy evaluation of the motion information collected in different sensors and the corresponding sensor position selection algorithm. Section 7 presents the experimental results, and we draw some conclusions in Section 8.

## 2. Related Work

### 2.1. Inertial Motion Capture

The inertial motion-capture systems include multiple inertial measurement units (IMUs) installed on various segments of the subject’s body, which can obtain motion measurements including acceleration, angular velocity and posture of body segments. Inertial motion-capture technology is relatively mature, and the device is portable, not affected by motion occlusion, and the data frame rate is high. Roetenberg et al. has proposed an inertial capture system based on Kalman filtering that can integrate 17 IMUs [4]. In order to improve the accuracy of inertial motion capture, related studies in recent years mainly focus on automatic calibration of inertial sensor installation parameters [12,13,14], motion reconstruction constraint conditions [15], and nonlinear optimization-based pose reconstruction methods [16].

Studies have shown that human motion includes a lot of redundant information and can be approximately presented by dimensions lower than the original degree of freedom of human motion [6,17,18], which inspired the study of sparse sensor motion capture. Some researchers developed motion-capture technologies combining sparse inertial sensors with video input [7,19] or optical reflective markers [20] or using only sparse optical markers [21]. Although these studies have achieved acceptable posture reconstruction performance, their applications are limited due to optical occlusion issues and high cost of use. In this study, we decided to use only commercially available IMUs for convenience without location measurements or a depth camera.

Existing research on human body posture reconstruction based on sparse inertial sensors mainly relies on motion prior data and models, including multilayer neural networks [10], local pose linear regression models [22], and deep-learning neural networks. A priori model of human motion based on a bidirectional recurrent neural network (Bi-RNN) using 5–6 IMUs to predict the body posture has been proposed [9]. According to the attitude estimation based on the prior model, von Marcard [8] et al. merged the prior model with inertial measurements, and proposed a motion reconstruction method based on offline optimization. At present, the research of motion-capture technology based on sparse inertial measurement is still in the preliminary stage. Several motion prior models have been proposed, but these models have limited accuracy for human body posture reconstruction in certain application scenarios.

### 2.2. Training Data Selection

Training data selection is an active research topic in machine learning. Through effective selection of training data, more informative samples are extracted, redundant samples and noise data are eliminated, to achieve better learning performance [23]. The current common training data selection methods include methods based on sampling, clustering, and information theory.

The sampling method is the widely adopted basic strategy to reduce the training set, which is simple, easy and fast. Some methods randomly selected training data according to the spatial nature of the sample [24,25,26]. Although they can effectively reduce the number of samples, the generalization ability of the obtained model cannot be guaranteed. Based on simple random sampling, the methods of uniformly and randomly selecting a compressed set were also studied [27,28,29].

Random selection of training samples cannot guarantee that the model works reliably in actual tasks. Therefore, scholars proposed to effectively select the training set by considering the distribution characteristics of the data, such as clustering methods. Zhai et al. presented an instance selection method based on supervised clustering, and the main idea is to select instances belonging to inner boundary and outer boundary of clusters [30]. Some researchers used k-means algorithm to partition the dataset into clusters and picked up data from each cluster [31,32]. While using k-means algorithms, the choice of the number of clusters is a challenging issue. In addition, the clustering results are sensitive to the choices of the initial points.

There are also methods using information theory to evaluate and select data samples. Zheng et al. applied a partial mutual information (PMI) technique to find the optimal dataset [33]. Liu et al. explored frame-level data selection based on the normalized frame-level entropy of Gaussian posterior probabilities obtained from the data [34]. The training data selected by the cross-entropy difference selection method proposed by Robert et al. has a good test performance and only requires a small amount of training data [35]. However, existing data selection methods are mainly used for the data reduction of large datasets to improve the computational efficiency of the general model training. When targeting certain application scenarios, researchers usually need to collect new datasets. Therefore, how to select IID training data for specific application scenarios from general datasets is a new topic that deserves further investigation.

### 2.3. Sensor Placement Selection

In existing research of sparse inertial sensors human motion reconstruction, sensor placement was usually manually decided, such as on the pelvis, head, forearms, and shanks [9]. In the past few years, some researchers have conducted research on the influence of sensor configuration on activity recognition. Cole et al. studied the choice of the position of the smart watch on the human wrist to predict smoking action [36]. Orha et al. compared the accuracy of placing the three-dimensional accelerometer on the right hand, right thigh, and chest to determine the best sensor placement based on the accuracy of the neural network classification of human activities [37]. On basis of this, some researchers have investigated more candidate positions for activity recognition tasks [38,39,40]. Banos et al. studied the influence of sensor placement on human motion recognition [41].

Instead of directly comparing the prediction accuracy of the sensor configurations, information-based techniques have been studied. These methods evaluated the value of sensor configurations without obtaining the testing performance in the final task. In this way, the searching efficiency can be improved. Kunze et al. determined the optimal sensor configuration based on mutual information between sensor data and the recognized action type [42], but this method did not consider redundancy within the sensor measurements. At present, the sensor configuration method for human activity recognition has been well studied, but there is still no related work for posture reconstruction. In addition, compared with the classification problem in activity recognition where there is only one output (i.e., action category), the output variables have multiple dimensions in the regression problem of human body posture reconstruction.

## 3. Method Overview

**Figure 1:**
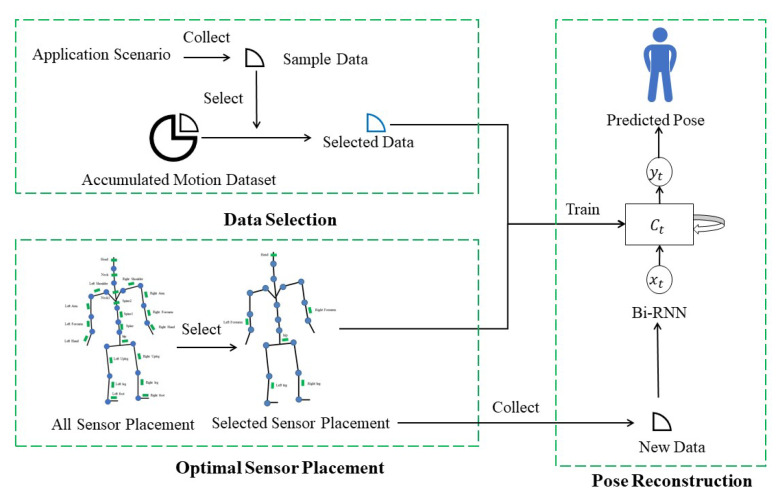
The workflow of data selection, optimal sensor configuration and pose reconstruction.

The current inertial motion-capture devices require the subject to wear about 17 sensors [4], and our goal is to reduce the sensor number while achieving acceptable performance in posture reconstruction accuracy. A deep neural network Bi-RNN is adopted as the basic model to map low-dimensional motion measurements to the whole-body posture. The measurements obtained from an IMU include the posture and acceleration of the sensor relative to the world coordinate system. Human bone kinematics are obtained based on installation parameters obtained through calibration. For better generalization, the input data are aligned with the orientation of the person based on the root bone (i.e., pelvis) orientation. The training of the Bi-RNN model requires a large amount of IID data of the target application scenario. To use the accumulated dataset and save time for transfer learning of posture reconstruction, we proposed a method to select useful training data that meet the IID condition from the accumulated motion dataset. At the same time, the position and number of sensors placed on the human body affect the performance of human posture reconstruction, so we also studied the optimal selection of the sensor placement to improve the reconstruction accuracy for target applications. An overview of the entire pipeline of the proposed method is shown in Figure 1. The upper left part of Figure 1 is the training data selection. We collect corresponding sample data according to specific application scenarios, and then select the data which has similar distribution to the sample data with small redundancy from the accumulated motion dataset. The bottom left part of Figure 1 is the optimal sensor configuration. We train the neural network on the right side of Figure 1 based on the selected training data and sensors. In actual use, the measurements of selected sensors is used as the input of the neural network, and finally the neural network outputs the posture.

## 4. Sparse Sensor Pose Reconstruction

### 4.1. Data Pre-Processing

In this study, inertial motion-capture suit Perception Neuron Studio is used to obtain human motion data [43]. Data obtained from the inertial sensors include the acceleration ${}^{W}a_{S}$ and rotation matrix ${}_{W}^{S}R$ of the sensor with respect to the world coordinate system *W*. To express the bone kinematics in *W* as (1), the kinematics of the sensors must be subjected to a set of calibrations wherein the orientation of sensor with respect to the bone is obtained. To identify the sensor to body alignment, the subject is asked to stand in a known pose for calibration. The rotation from sensor to body ${}_{S}^{B}R$ is determined by matching the orientation of the sensor in the world coordinate system ${}_{W}^{S}R$ with the known orientation of each bone ${}_{W}^{B}R$ in this pose.

(1) $${}_{W}^{B}R{=}_{W}^{S}R\cdot_{S}^{B}R$$

For better generalization, the input data are normalized with the person’s orientation. Then, we standardized all bone orientations with respect to the root bone, which is the pelvis. ${}_{w}^{Root}R$ denotes the orientation of the root bone with respect to the world coordinate system, and ${}_{w}^{Root}a$ denotes the acceleration of the root with respect to world coordinate system. The normalized orientation and acceleration of each bone are obtained as (2) and (3).

(2) $${}_{Root}^{B}R{=}_{Root}^{W}R\cdot_{W}^{B}R$$

(3) $${}^{Root}a_{B}{=}_{Root}^{W}R\cdot {(}^{W}a_{B}{-}^{W}a_{Root})$$

### 4.2. Bi-RNN Model

**Figure 2:**
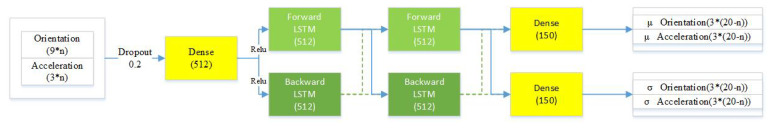
The structure of the Bi-RNN, and *n* is the sparse sensor number.

Human body motion pattern has temporal characteristics, and the pose of the current frame is often correlated with history and future motion data. Bidirectional Recurrent Neural Network (Bi-RNN) is suitable to learn the temporal relationship between history and future pose data and the current frame pose [44], so Bi-RNN is adopted in this study. The basic structure of our Bi-RNN model refers to the work done by Huang et al. [9], which is shown in Figure 2. Given a training dataset $D={\left\{\left(x^{i},y^{i}\right)\right\}}_{i=1}^{N}$ including N data frames, the objective is to train a f:x→y function which can predict the distribution of the kinematic data of unselected sensors *y* from the measurements of selected sensors *x*. Huang et al. tried to fit the model to predict the human body Skinned Multi-Person Linear Model (SMPL). SMPL is a parametrized model of 3D human body and pose that takes 72 pose parameters and 10 shape parameters, and returns a mesh with 6890 vertices. Unlike Huang et al. that train the model to predict both pose and shape parameters, we directly predict the body posture. Numbers in brackets in Figure 2 are the input dimensions, output dimensions and number of units in the respective layer. The dropout probability of the input layer is 0.2. The middle layer is composed of Long Short-Term Memory (LSTM) units, and the sequence length of Bi-RNN is 300 frames including 150 frames of both past and future data.

The inputs of the Bi-RNN are the acceleration vector and orientation in rotation matrix format of selected bones, and the outputs are the acceleration norm and orientation in axis angle format of all other unselected bones. Through comparison of the reconstruction performance using different orientation formats in the Bi-RNN, the combination of rotation matrix input and axis angle output achieves the highest accuracy in the experiments.

## 5. Training Data Selection

### 5.1. Problem Formulation

Conventionally, the entire available dataset is used for deep neural network training. However, there may be redundant data or irrelevant data, which may affect the training performance of the model for specific application scenarios. For example, when learning motion model for walk reconstruction, the motion-capture data of other types of activities, such as run, jump, and dance, are irrelevant and may not contribute to the reconstruction performance of walk. In addition, human walk is a repetitive movement consisting of consecutive similar gait cycles, and a long sequence of normal walk data with consistent pattern may have large redundancy, which will only increase the training time of the model. Therefore, selecting appropriate training data according to the target scenario is desirable to improve the accuracy of pose reconstruction and reduce model training time.

We denote the accumulated dataset as $D_{A}$ and the motion feature distribution within the dataset can be different from the target application scenario. To select IID data for a specific application scenario from the accumulated dataset, we first collect a small amount of reference sample data $D_{Ref}$ in this scenario and estimate its a priori distribution of movements features. The goal of training data selection is to re-sample continuous data segments from $D_{A}$ to obtain actual training data *D* that has similar distribution to the reference data with small redundancy. Then, the selected training data and reference data are used for the Bi-RNN pose reconstruction model training. The training data selection problem can be formulated into an optimization problem as (4). Here, *D* is the selected data, which has *K* continuous segments with various lengths. H(D) is the amount of information of the selected data, i.e., the entropy of *D*, $d_{DP}$ evaluates the distribution difference between *D* and $D_{Ref}$, and N(D) means the amount of data in *D*. The objective is to select adequate data with enough information for the target scenario.

(4) $$\underset{D\subseteq D_{A}}{max}\;\alpha H\left(D\right)-\beta d_{DP}\left(D,D_{Ref}\right)-\gamma N\left(D\right),$$

**Figure 3:**
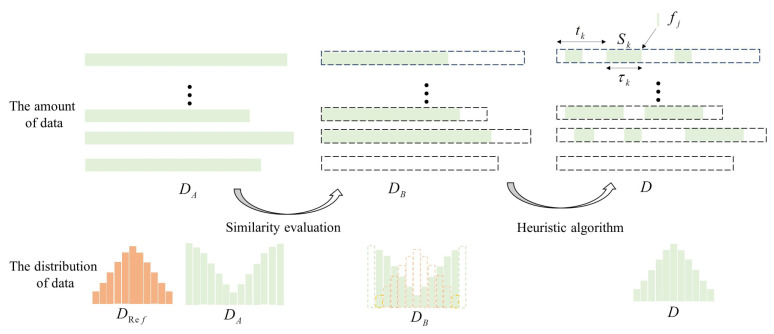
Illustration of the training data selection process. The upper part of the figure is the data segments selected during the procedure. The lower part of the figure shows the data distribution in histograms of both reference data and selected data. In this first step of similar data selection, the selected data segments share the same range of values with the reference data. In the second step of heuristic algorithm of data selection optimization, the selected data segments tend to have similar distribution as the reference data.

Assuming *D* includes *K* motion data segments and $D_{A}$ includes *M* motion data segments, D can be expressed as $\left\{S_{k}\right\},k\in \left[1,K\right]$ where $S_{k}=\left[m_{k},t_{k},\tau_{k}\right]$ representing a data segment selected from $m_{k}^{th}$ data segment from $D_{A}$. $S_{k}=\left\{f_{j}\right\},j\in \left[1,\tau_{k}\right]$ starts from the $t_{k}^{th}$ frame and takes $\tau_{k}$ frames in total. Every data frame $f_{j}$ contains $N_{f}$ features denoted by $\left\{\theta_{i}\right\},i\in \left[1,N_{f}\right]$. Because the value function is non-derivable and discontinuous, it is challenging to solve this problem from the perspective of theoretical analysis. Thus, a heuristic algorithm is proposed to solve the problem into two steps. First, a similar and continuous dataset $D_{B}\subseteq D_{A}$ is chosen from the dataset using cosine angle to evaluate the difference between the chosen data and the reference data. In this way, we can delete irrelevant motion data from $D_{A}$, reducing the search space of heuristic algorithm. Second, we select data with distribution similar to the reference data from $D_{B}$ by adopting a heuristic algorithm to find an approximate optimum value of the optimization problem. An illustration of the data selection process is shown in Figure 3.

### 5.2. Similar Data Selection

The data selection problem is equivalent to determining a Boolean flag of selected or not for every data frame in the whole dataset. Since the dataset may have a huge number of frames inside, the variable state space to be optimized can be huge as well. Considering that continuous pieces of data segments are preferred in the data selection for Bi-RNN training, we divide each complete segment of data in $D_{A}$ into data units with a length of N=50 frames (i.e., 0.5 s) to lower down the state space dimension. To select data following the IID condition of the target application scenario, we designed a two-step efficient algorithm. First, we select data units from the whole dataset that are similar to any piece of the reference data unit in terms of motion feature values. The selected data are regarded as the similar dataset $D_{B}$. The similar dataset further narrows down the state space of optimal training data searching.

In the implementation, the cosine angle metrics is used to evaluate the similarity between a selected data unit $u_{1}$ in $D_{A}$ and a reference data unit $u_{2}$ in $D_{Ref}$ as (5). We calculate the similarity between each pair of $u_{1}$ and $u_{2}$, and data units $u_{1}$ whose maximum similarity with any reference data unit $\eta_{max}\left(u_{1},u_{2}\right)\geq \eta_{0}$ are selected into $D_{B}$. Since the input sequence length of Bi-RNN is L=300, only data segments with more than *L* frames are selected. If $\eta_{0}$ is too large, the searching space will be too large; if $\eta_{0}$ is too small, some useful data will be filtered out. Through experimental tuning, $\eta_{0}=0.8$ is chosen in this study.

(5) $$\eta \left(u_{1},u_{2}\right)=\frac{1}{N}\sum_{i=1}^{N}\frac{\langle x_{1i},x_{2i}\rangle }{∥x_{1i}∥∥x_{2i}∥},x_{1i}\in u_{1},x_{2i}\in u_{2},$$

### 5.3. Optimal Data Selection

After $D_{B}$ is selected from the original dataset $D_{A}$, we need to further select training data $D\subseteq D_{B}$ to follow the IID condition of the target scenario and be as continuous as possible, contain more information, and has small redundancy at the same time. This optimization problem has been introduced in (4). IID condition requires that the joint distribution of the motion features of the selected training data *D* should be the same as the reference data $D_{Ref}$. However, evaluating the similarity of the joint distribution of motion features is difficult due to the high dimensionality of the data. When the motion data features of both *D* and $D_{B}$ are Gaussian and have similar value range (guaranteed by similar data selection), and the marginal probability distributions of each feature are similar, then the joint probability distribution should be similar.

The Kullback–Leibler (KL) divergence is a measure of the difference between one probability distribution and another. We use the KL divergence to evaluate the marginal distribution difference between each feature of *D* and $D_{Ref}$, and obtain the overall distribution distance as (6). Here, $N_{f}$ is the number of features, $P_{i}\left(x\right)$ is the probability distribution of the $i^{th}$ feature of *D*, $Q_{i}\left(x\right)$ is the probability distribution of the $i^{th}$ feature of reference data. To discretize each feature to better express its distribution, we divide the value range of every feature into 20 intervals χ according to the maximum and minimum values of each feature to calculate its probability distribution. The calculation of $P_{i}\left(x\right)$ is given as (7) and (8). $N_{i}\left(x\right)$ counts the number of data falling into the data interval of x∈χ. Similarly, $Q_{i}\left(x\right)$ can be calculated.

(6) $$d_{DP}\left(D,D_{Ref}\right)=\frac{1}{N_{f}}\sum_{i=1}^{N_{f}}\sum_{x\in \chi }P_{i}\left(x\right)log\frac{P_{i}\left(x\right)}{Q_{i}\left(x\right)},$$

(7) $$P_{i}\left(x\right)=\frac{\sum_{k=1}^{K}\sum_{j=1}^{l_{k}}\delta_{kji}\left(x\right)}{\sum_{k=1}^{K}l_{k}},i\in \left[1,N_{f}\right],$$

(8) $$\delta_{kji}=\left\{\begin{array}{cc} 1, & if\;\theta_{kji}\in x,\\ 0, & otherwise. \end{array}\right.$$

In this paper, we use information entropy to evaluate the amount of information of the data as (9). In the same way, we divide the features into 20 intervals x∈χ according to the maximum and minimum values of each feature to calculate the probability distribution of each feature. N(D) is the number of data we selected given in (10), which needs to be minimized to remove redundant data.

(9) $$H\left(D\right)=\frac{1}{N_{f}}\sum_{i=1}^{N_{f}}\sum_{x\in \chi }P_{i}\left(x\right)logP_{i}\left(x\right).$$

(10) $$N\left(D\right)=log\left(\sum_{k}l_{k}\right).$$

In summary, the cost function of the training data selection optimization problem can be rewritten as (11). Considering maximizing the information term, it is desirable to choose the data distribution P similar to Q and preferably uniformly distributed. When considering minimizing the data amount, data with fewer segments and frames are preferred. Because *D* is discontinuous and $f_{ds}\left(D\right)$ is non-derivable, it is difficult to find the optimal value of $f_{ds}$ from the perspective of theoretical analysis, so we propose a heuristic searching algorithm. To solve the optimization problem, a greedy algorithm is adopted to break down the original problem into a series of sub-problems, and in each iteration, we try to identify the optimal data segment that can be deleted from the existing selected dataset. Initially, set $D=D_{B}$ which can be expressed as $\left\{S_{k_{B}}\right\}$, $k_{B}\in \left[1,K_{B}\right]$. To delete the first data segment $S_{k_{B},0}$, we need to consider the $f_{ds}$ value and the data segment satisfies (12) will be deleted. The next data segment $S_{k_{B}}^{*}$ to be deleted is the data segment satisfying (13). Equation (13) is iterated until $f_{ds}\left(D\backslash S_{k_{B}}^{*}\right)$ converges to a local minimum.

(11) $$\underset{D\subseteq D_{B}}{max}\;f_{ds}\left(D\right)=\frac{\alpha }{N_{f}}\sum_{i=1}^{N_{f}}\sum_{x\in \chi }P_{i}\left(x\right)\left[log\left(P_{i}^{\alpha }\left(x\right)\right)-log\left(\frac{P_{i}^{\beta }\left(x\right)}{Q_{i}^{\beta }\left(x\right)}\right)\right]-log\left(\sum_{k}l_{k}\right)$$

(12) $$S_{k_{B},0}=\underset{S_{k_{B}},k_{B}\in \left[1,K_{B}\right]}{\arg min}\;f_{ds}\left(S_{k_{B}}\right)$$

(13) $$S_{k_{B}}^{*}=\underset{S_{k_{B}}\subseteq D,k_{B}\in \left[1,K_{B}\right]}{\arg max}\;f_{ds}\left(D\backslash S_{k_{B}}\right)$$

## 6. Optimal Sensor Placement

### 6.1. Problem Formulation

The purpose of the sensor selection is to find a sparse sensor configuration that uses fewer sensors and has high body posture reconstruction accuracy. Generally, when selecting sensors, only the amount of information that can be measured by different sensors is considered. However, our objective is to estimate the measurements of the unselected sensors from the measurements of the selected sensors, thus, we need to maximize the correlation between the measurements of the selected sensors and the unselected sensors. Due to correlation between the sensors, the selected sensors will also contain related measurements. Traditional information-based feature selection methods usually only consider the correlation between the selected sensors and the unselected sensors. In this way, the sensors with more information redundancy are usually selected at the same time, and the sensor with less correlation with the unselected sensors but no redundancy is usually ignored. This is actually not preferred.

In order that the selected sensors can measure as much body motion information as possible, this paper draws on the idea of Max-Relevance and Min-Redundancy (mRMR) feature selection method in sensor selection [45]. It not only considers the relevance of the candidate sensor and the unselected sensors, but also considers the redundancy among the candidate sensor and the selected sensors. In this section, we introduce the relevance evaluation of the candidate sensor and the unselected sensors, and the measurement of the redundancy between the candidate sensor and the selected sensors. Then, we determine the optimal sensor placement based on the proposed metrics.

**Figure 4:**
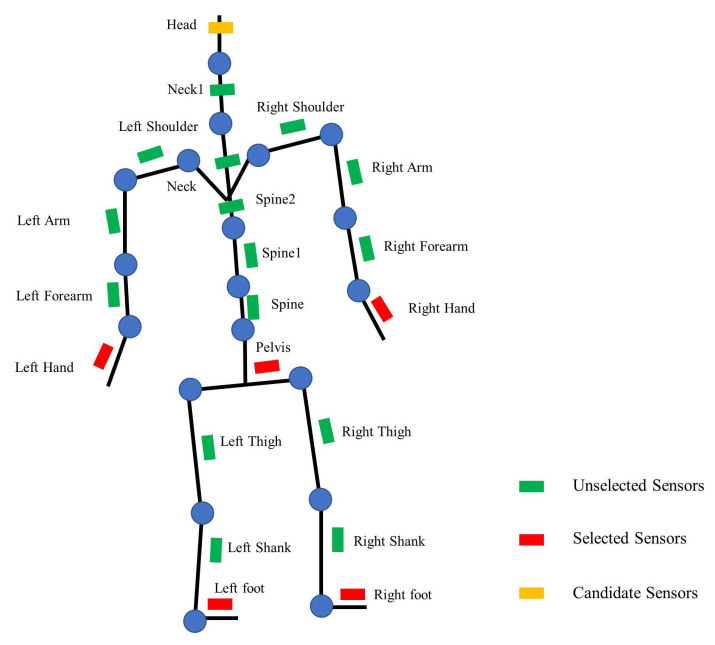
Available positions of inertial sensors in an inertial motion-capture system.

All available sensor placements in an inertial motion-capture system are shown in Figure 4. There are a total of 21 positions to be selected, including hands, forearms, arms, thighs, shanks, feet, shoulders, spine, root bone, head and neck. Because it is necessary to calculate the relative posture and acceleration of each bone to the root bone, a sensor placed at the root bone (i.e., pelvis) is required.

### 6.2. Maximum Information Coefficient

The maximum information coefficient (MIC) proposed by Reshef et al. can evaluate most of the relationships between two sets of data, including linear and nonlinear relationships [46]. Compared with other coefficients, MIC has the characteristics of universality, fairness and symmetry, and can accurately evaluate the connection between various sensor measurements. The idea of MIC is to discretize variables in a two-dimensional space based on the relationship between two variables and use a scatter plot to represent them. The coefficient will divide the two-dimensional space into a certain number of intervals in the x and y directions, and then check how the current scatter points fall into each grid. Equation (14) gives the definition of the MIC, where I(X,Y) is the mutual information between two variables X and Y, *a*, *b* are the number of grids divided in the *x*, *y* directions, and *B* is about 0.6 power of the amount of data.

(14) $$MIC\left(x;y\right)=\underset{|a||b|<B}{max}\frac{I(x;y)}{log_{2}\left(min(|a|,|b|)\right)}$$

### 6.3. Max-Relevance and Min-Redundancy

Suppose there are sensor position X and sensor position Y, and the dimension of measurement feature of each sensor is s, then the correlation between the two sensor position measurements is defined as (15). We sum up the MIC between each feature in the measurements of sensor position X and Y to obtain the correlation value.

(15) $$Rele\left(x;y\right)=\sum_{i=1}^{s}\sum_{j=1}^{s}MIC\left(x_{i};y_{j}\right)$$

We use Max-Relevance and Min-Redundancy (mRMR) to evaluate the measurement information quality of a sensor group. Then, the purpose of optimal sensor selection is to find a sensor group that has greater correlation with the unselected sensors and less redundancy among the selected sensors. First, we find the first sensor with the greatest correlation with all unselected sensors based on the principle of maximum correlation. Second, we use the principle of maximum correlation and minimum redundancy to find the sensor with the largest correlation with the unselected sensor minus the redundancy with the selected sensor as the next sensor to be chosen. This iteration is repeated until the number of sensors and the accuracy of posture reconstruction meet the requirements.

Specifically, the purpose of maximum correlation is to find a sensor group S with the highest correlation with the unselected sensor group C. We add up the correlations between all selected sensors and unselected sensors, and divide by the number of selected and unselected sensors for normalization. The correlation between selected sensors and unselected sensors is defined as (16). On the other hand, the redundancy between sensors in the selected sensor group is defined as (17). The smaller the value of R, the lower the redundancy between sensors. In combination with the correlation and redundancy, the criteria for evaluating the information quality of a sensor group is defined as (18). The larger Φ is, the larger the amount of information can be obtained by the sensor group measurements in the posture reconstruction task.

(16) $$maxT\left(S,C\right),\;T=\frac{1}{\left|S||C\right|}\sum_{x_{i}\in S}\sum_{c_{j}\in C}Rele\left(x_{i};c_{j}\right)$$

(17) $$minR\left(S\right),\;R=\frac{1}{{\left|S\right|}^{2}}\sum_{x_{i}\in S}\sum_{x_{j}\in S}Rele\left(x_{i};x_{j}\right)$$

(18) maxΦ(T,R),Φ=T−R

### 6.4. Sensor Selection

A greedy algorithm is adopted to find the optimal solution of Φ. When selecting the first sensor, only the correlation between the sensor and the unselected sensor is considered. The greater the correlation, the more important the sensor is for measuring body motion information. We calculate the correlation between each sensor and the unselected sensor through the previous definition and choose the most relevant as the first selected sensor. For better computational efficiency, incremental search method is used based on mRMR to find the approximate optimal sensors. Assuming that M−1 sensor groups $S_{m-1}$ have already been chosen, the goal is to find the $m^{th}$ sensor from the unselected sensors $\left\{S_{0}-S_{m-1}\right\}$ to maximize Φ. Correspondingly, the incremental algorithm needs to optimize the objective function (19).

(19) $$\underset{x_{j}\in X-S_{m-1}}{max}\left(\frac{1}{\left|C\right|}\sum_{x_{i}\in S}\sum_{c_{j}\in C}Rele\left(x_{i};c_{j}\right)-\frac{1}{\left|S\right|}\sum_{x_{i}\in S}\sum_{x_{j}\in S}Rele\left(x_{i};x_{j}\right)\right)$$

## 7. Experiment

### 7.1. Data Collection and Model Training

In the experiments, we collected motion data in daily activities and sports, including walking, running and jumping, playing basketball, football, table tennis, and so on. The entire dataset contains about 800,000 frames of motion data of 4 people with a total length of 2.5 hA total of 20,000 frames of walking, running, and playing basketball are selected as test set, and the rest data are regarded as the training set. On average, the Bi-RNN model was trained on GPU RTX 2080 for about 6 h until convergence.

### 7.2. Data Selection Algorithm Performance

To verify the performance of data selection algorithm, we train the model separately with the entire dataset, manually selected data, and the data selected by the proposed data selection algorithm, and then test in the corresponding motion of the test set. In this case, the sensor configuration is the default 6 sensor configuration including head, forearms, shanks and pelvis. The pose reconstruction error and the number of data frames used for model training are shown in Table 1. The pose reconstruction error refers to the relative rotation angle between the reconstructed pose and the actual pose. The manual data selection assumes that the person already knows the type of movement of the dataset, and manually selects the corresponding labeled data files as the training data. The general model is trained by the entire dataset. As the results, the model trained on the algorithm-selected data achieves the best performance in the posture reconstruction accuracy in all the motion types. The model trained on the entire dataset performs better than the model trained on manually selected data.

Moreover, the amount of data selected by our data selection algorithm is less than all the data, but higher than the amount of data selected manually. This indicates that in addition to data files with corresponding labels, other data files of other motion types may also contain useful motion data that are similar to target motion type. Our data selection algorithm can extract these effective data segments from different test files across the entire dataset. The smaller amount of data selected by the proposed algorithm significantly reduces the training time of the model to less than 1/3, compared to the entire dataset.

### 7.3. Sensor Selection Algorithm Performance

**Figure 5:**
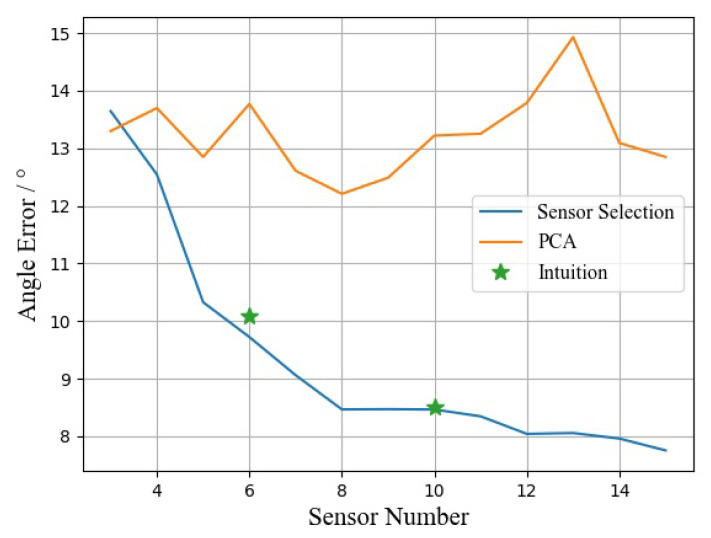
Curve of error and sensor number.

To validate the effectiveness of the proposed sensor selection algorithm, we test the performance of the algorithm against some other sensor selection methods using the entire dataset. We compare our method with the modified PCA [47] for feature selection. The modified PCA drawn from the PCA of the input data to leverage the relative importance of the principal components along with the coefficients within the principal directions of the data to uncover the ranking of the features and select the top few features. the relationship curve between the average posture reconstruction angular error and the number of sensors (including the root bone) are shown in Figure 5. In addition, we manually selected the default 6 sensors and 10 sensors configuration (hands, arms, thighs, feet, pelvis and head) by experiences as ‘intuition’ and evaluated its performance as well. It is shown that our sensor selection algorithm outperforms PCA and at the same time more practical and automated than the default manual sensor configuration. As the number of sensors increases, the pose reconstruction error of our sensor selection algorithm gradually decreases, while PCA fluctuates around a certain value. This may be because PCA did not consider the information redundancy between the sensors, resulting in that the information measured by the sensors selected later cannot reduce the posture reconstruction error.

### 7.4. Performance of Combinations

**Figure 6:**
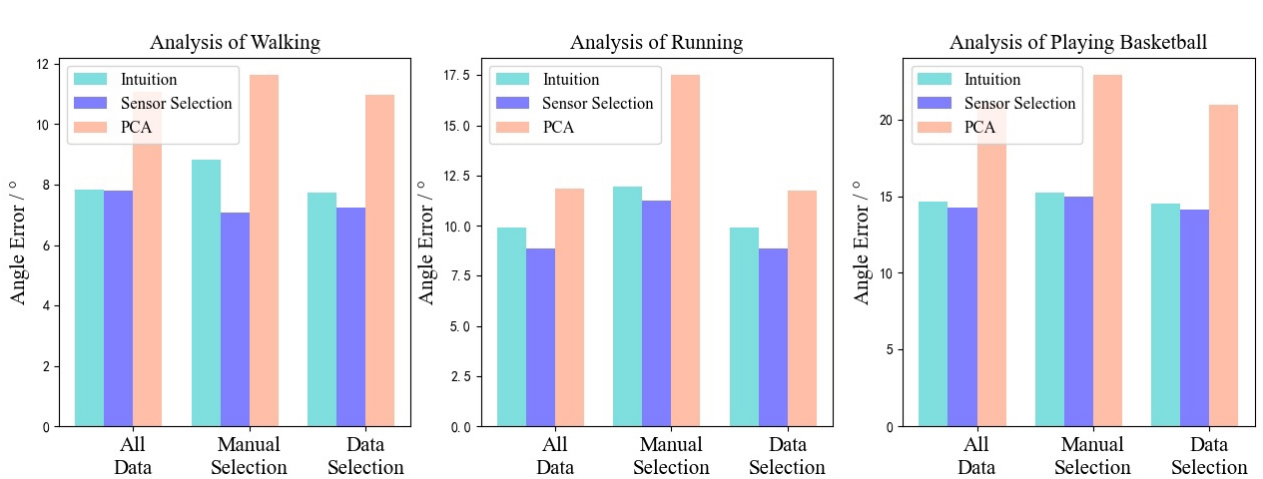
Comprehensive evaluation of algorithm performance.

To evaluate the performance of combinations of both training data selection and sensor placement selection, we further conducted comprehensively comparison study on 6 sensors configuration of different motion types. The experimental results are shown in Figure 6. In the figure, we can see that the algorithm-based sensor selection results is better than ‘intuition’ and PCA in most cases. The accuracy of the model trained by the proposed data selection method is also better than the model trained on the entire dataset and manually selected data across different motion types.

### 7.5. Pose Estimation Evaluation

**Figure 7:**
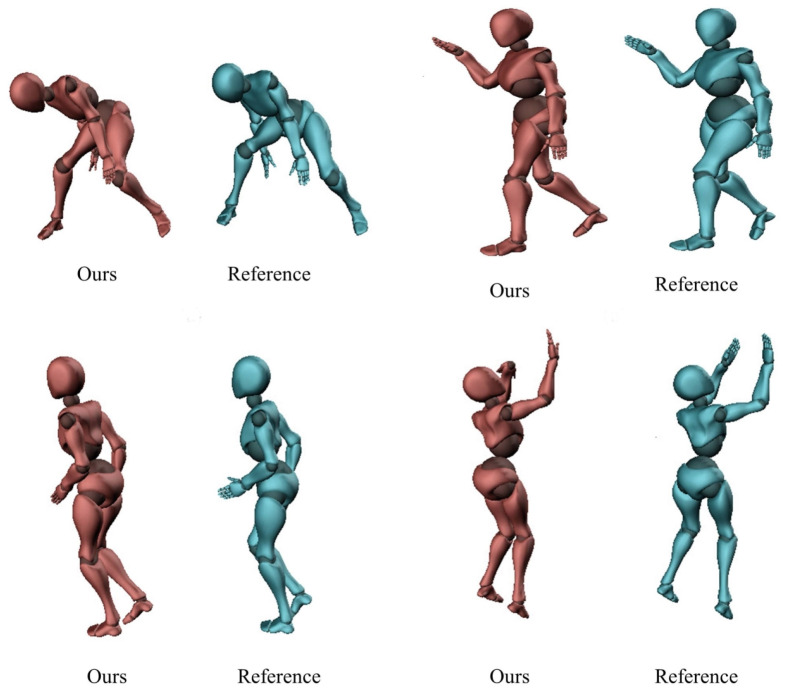
Posture reconstruction results of some motion frames of playing basketball with optimal 6 sensor configuration.

The proposed method has a good posture reconstruction performance for regular exercise reconstructed frames, such as simple walking and running. Table 1 summarizes the posture reconstruction accuracy among different motion types. Figure 7 shows the posture reconstruction performance of some motion frames of playing basketball with obtained optimal 6 sensor configuration. The characters in mixamo [48] are used in the production of the character animation in Figure 7. For some complex sports, such as playing basketball in Figure 7, there are defects in the hand posture. For regular motions, the neural network can easily learn the movement patterns. The complex movements have patterns with more variations, and the amount of complex motion data contained in the training dataset is also limited compared to the regular motions. Thus, the complex motion posture reconstruction performance is worse than simple motion.

## 8. Conclusions

In this work, we propose methods for training data selection and sensor position selection in sparse inertial sensor human posture reconstruction under specific application scenarios. The problem of deep-learning training data selection is formulated into an optimization problem. We present a two-step information-based heuristic data selection algorithm by selecting similar data and optimal data from the entire dataset with respect to the reference data collected from the target scenario. Compared with model trained on the entire dataset and manually selected data, the Bi-RNN model trained on the algorithm-selected data have the better performance and less training time.

On the other hand, the sparse sensor position selection is also studied to exploit most information from partial observation of human movement. A greedy algorithm is proposed to search a relative optimum sensor group for maximum motion information and minimum redundancy. Experimental results show that the posture reconstruction performance of the sensor configuration selected by our sensor selection algorithm is better than both PCA and the manually picked default sensor configuration. As the number of sensors increases, the pose reconstruction error of our sensor selection algorithm gradually decreases, which proves that the proposed algorithm takes into account the advantages of sensor measurement information redundancy.

Currently, we proposed heuristic algorithms with greedy strategy to obtain approximate solutions of the established optimization problem. In the future, we will further investigate optimization methods for both training data selection and sensor position selection from a theoretical point of view. Moreover, we will also conduct research on the enhancement of deep neural network structure and the integration of motion analysis for sparse inertial sensor posture construction tasks.

## Figures and Tables

**Table 1:** Motion Reconstruction Errors of Models with Default 6 Sensors Configuration.

Motion Type	All Data	Manual Selected Data	Algorithm Selected Data
Walk	7.83° (787,000 frames)	8.84° (98,000 frames)	**7.74**° (170,000 frames)
Run	9.92° (787,000 frames)	11.94° (63,000 frames)	**9.88**° (210,000 frames)
Play Basketball	14.64° (787,000 frames)	15.22° (92,000 frames)	**14.52**° (206,000 frames)

## Data Availability

Data sharing not applicable.

## Abbreviations

The following abbreviations are used in this manuscript:

IID	Independent Identical Distribution
MIC	Mutual Information Theory
PCA	Principal Component Analysis
mRMR	Max-Relevance and Min-Redundancy
Bi-RNN	Bidirectional Recurrent Neural Network
LSTM	Long Short-Term Memory

## References

[1] Muñoz J.E., Chavarriaga R., Villada J.F., SebastianLopez D. BCI and motion capture technologies for rehabilitation based on videogames. Proceedings of the IEEE Global Humanitarian Technology Conference (GHTC 2014).

[2] Jie G., Guiling C., Lin H., Dong Z. Application research on motion capture system data reuse in virtual reality environment. Proceedings of the 2010 Second International Conference on Intelligent Human-Machine Systems and Cybernetics.

[3] Noiumkar S., Tirakoat S. Use of optical motion capture in sports science: A case study of golf swing. Proceedings of the 2013 International Conference on Informatics and Creative Multimedia.

[4] Roetenberg D., Luinge H., Slycke P. (2009). Xsens Mvn: Full 6 Dof Human Motion Tracking Using Miniature Inertial Sensors. Xsens Motion Technologies BV. Technical Report. http://human.kyst.com.tw/upload/pdfs120702543998066.pdf.

[5] Troje N.F. (2002). Decomposing biological motion: A framework for analysis and synthesis of human gait patterns. J. Vis..

[6] Sanger T. (2000). Human arm movements described by a low-dimensional superposition of principal components. J. Neurosci. Off. J. Soc. Neurosci..

[7] Safonova A., Hodgins J., Pollard N. (2004). Synthesizing physically realistic human motion in low-dimensional, behavior-specific spaces. ACM Trans. Graph..

[8] Marcard T., Rosenhahn B., Black M., Pons-Moll G. (2017). Sparse inertial poser: Automatic 3D human pose estimation from sparse iMUs. Computer Graphics Forum.

[9] Huang Y., Kaufmann M., Aksan E., Black M.J., Hilliges O., Pons-Moll G. (2019). Deep inertial poser: Learning to reconstruct human pose from sparse inertial measurements in real time. ACM Trans. Graph..

[10] Wouda F., Giuberti M., Bellusci G., Veltink P. (2016). Estimation of full-bod poses using only five inertial sensors: An eager or lazy learning approach?. Sensors.

[11] Bertsekas D.P., Tsitsiklis J.N. (2008). Introduction to Probability.

[12] Bouvier B., Duprey S., Claudon L., Dumas R., Savescu A. (2015). Upper limb kinematics using inertial and magnetic Sensors: Comparison of Sensor-to-Segment calibrations. Sensors.

[13] Taetz B., Bleser G., Miezal M. Towards self-calibrating inertial body motion capture. Proceedings of the 2016 International Conference on Information Fusion (FUSION).

[14] Müller P., Bégin M., Schauer T., Seel T. (2017). Alignment-free, self-calibrating elbow angles measurement using inertial sensors. IEEE J. Biomed. Health Inform..

[15] Miezal M., Taetz B., Bleser G. Real-time inertial lower body kinematics and ground contact estimation at anatomical foot points for agile human locomotion. Proceedings of the 2017 IEEE International Conference on Robotics and Automation (ICRA).

[16] Kok M., Hol J.D., Schön T.B. (2014). An optimization-based approach to human body motion capture using inertial sensors. IFAC Proc. Vol..

[17] Vlasic D., Adelsberger R., Vannucci G., Barnwell J., Gross M., Matusik W., Popović J. (2007). Practical motion capture in everyday surroundings. ACM Trans. Graph..

[18] Trumble M., Gilbert A., Hilton A., Collomosse J. Deep autoencoder for combined human pose estimation and body model upscaling. Proceedings of the European Conference on Computer Vision (ECCV).

[19] Pons-Moll G., Baak A., Gall J., Leal-Taixe L., Muller M., Seidel H.P., Rosenhahn B. Outdoor human motion capture using inverse kinematics and von mises-fisher sampling. Proceedings of the 2011 International Conference on Computer Vision.

[20] Marcard T., Pons-Moll G., Rosenhahn B. (2016). Human pose estimation from video and IMUs. IEEE Trans. Pattern Anal. Mach. Intell..

[21] Andrews S., Huerta I., Komura T., Sigal L., Mitchell K. Real-time physics-based motion capture with sparse sensors. Proceedings of the 13th European conference on visual media production (CVMP 2016).

[22] Liu H., Wei X., Chai J., Ha I., Rhee T. Realtime human motion control with a small number of inertial sensors. Proceedings of the Symposium on Interactive 3D Graphics and Games.

[23] Yang Y., Lu Y., Yao M., Xia Y., Hao Y. Training data selection for short term load forecasting. Proceedings of the 2011 Third International Conference on Measuring Technology and Mechatronics Automation.

[24] Balcázar J., Dai Y., Watanabe O., Abe N., Khardon R., Zeugmann T. (2001). A random sampling technique for training support vector machines. Algorithmic Learning Theory.

[25] Ferragut E.M., Laska J. Randomized sampling for large data application of SVM. Proceedings of the 2012 11th International Conference on Machine Learning and Applications.

[26] Tsagalidis E., Evangelidis G. The effect of training set selection in meteorological data mining. Proceedings of the 2010 14th Panhellenic Conference on Informatics.

[27] Lee Y.J., Mangasarian O.L. RSVM: Reduced support vector machines. Proceedings of the 2001 SIAM International Conference on Data Mining.

[28] Lee Y., Huang S. (2007). Reduced support vector machines: A statistical theory. IEEE Trans. Neural Netw..

[29] Li X., Cervantes J., Yu W. (2012). Fast classification for large data sets via random selection clustering and support vector machines. Intell. Data Anal..

[30] Zhai J.H., Xui H.Y., Zhang S.F., Li N., Li T. Instance selection based on supervised clustering. Proceedings of the 2012 International Conference on Machine Learning and Cybernetics.

[31] Koskimäki H., Juutilainen I., Laurinen P., Röning J. Two-level clustering approach to training data instance selection: A case study for the steel industry. Proceedings of the 2008 IEEE International Joint Conference on Neural Networks.

[32] Kaneda Y., Zhao Q., Liu Y. On-line training with guide data: Shall we select the guide data randomly or based on cluster centers?. Proceedings of the 2016 IEEE Symposium Series on Computational Intelligence (SSCI).

[33] Zheng J., Yang W., Li X. Training data reduction in deep neural networks with partial mutual information based feature selection and correlation matching based active learning. Proceedings of the 2017 IEEE International Conference on Acoustics, Speech and Signal Processing (ICASSP).

[34] Liu S.H., Chu F.H., Lin S.H., Lee H.S., Chen B. Training data selection for improving discriminative training of acoustic models. Proceedings of the 2007 IEEE Workshop on Automatic Speech Recognition Understanding (ASRU).

[35] Moore R., Lewis W. Intelligent selection of language model training data. Proceedings of the 48th Annual Meeting of the Association for Computational Linguistics.

[36] Cole C.A., Thrasher J.F., Strayer S.M., Valafar H. Resolving ambiguities in accelerometer data due to location of sensor on wrist in application to detection of smoking gesture. Proceedings of the 2017 IEEE EMBS International Conference on Biomedical Health Informatics (BHI).

[37] Orha I., Oniga S. Study regarding the optimal sensors placement on the body for human activity recognition. Proceedings of the 2014 IEEE 20th International Symposium for Design and Technology in Electronic Packaging (SIITME).

[38] Dobrucalı O., Barshan B. (2013). Sensor-activity relevance in human activity recognition with wearable motion sensors and mutual information criterion. Information Sciences and Systems 2013.

[39] Ching Y.T., Cheng C.C., He G.W., Yang Y.J. (2017). Full model for sensors placement and activities recognition. Proceedings of the UbiComp ’17 2017 ACM International Joint Conference on Pervasive and Ubiquitous Computing and Proceedings of the 2017 ACM International Symposium on Wearable Computers.

[40] Murao K., Mogari H., Terada T., Tsukamoto M. Evaluation function of sensor position for activity recognition considering wearability. Proceedings of the 2013 ACM Conference on Pervasive and Ubiquitous Computing Adjunct Publication.

[41] Banos O., Toth M.A., Damas M., Pomares H., Rojas I. (2014). Dealing with the effects of sensor displacement in wearable activity recognition. Sensors.

[42] Kunze K., Lukowicz P. (2014). Sensor placement variations in wearable activity recognition. IEEE Pervasive Comput..

[43] (2020). Perception Neuron Studio System Landing Page. https://neuronmocap.com/perception-neuron-studio-system.

[44] Bahdanau D., Cho K., Bengio Y. Neural machine translation by jointly learning to align and translate. Proceedings of the 2015 International Conference on Learning Representations (ICLR).

[45] Ding C., Peng H. (2005). Minimum redundancy feature selection from microarray gene expression data. J. Bioinform. Comput. Biol..

[46] Reshef D.N., Reshef Y.A., Finucane H.K., Grossman S.R., McVean G., Turnbaugh P.J., Lander E.S., Mitzenmacher M., Sabeti P.C. (2011). Detecting novel associations in large data sets. Science.

[47] Malhi A., Gao R. (2004). PCA-based feature selection scheme for machine defect classification. IEEE Trans. Instrum. Meas..

[48] Mixamo (2020). https://www.mixamo.com/#/?page=1&type=Character.

